# Complete Genome Sequence of *Maize Mosaic Nucleorhabdovirus*

**DOI:** 10.1128/MRA.00637-19

**Published:** 2019-07-18

**Authors:** Kathleen M. Martin, Anna E. Whitfield

**Affiliations:** a Department of Entomology and Plant Pathology, North Carolina State University, Raleigh, North Carolina, USA; Portland State University

## Abstract

The complete genome sequence of maize mosaic virus (MMV) was obtained using next-generation sequencing from infected Peregrinus maidis and rapid amplification of cDNA ends from infected Zea mays. The genome of MMV is 12,170 bases, and this project completed the 5′ and 3′ ends and amended the polymerase sequence.

## ANNOUNCEMENT

*Maize mosaic nucleorhabdovirus* is a negative-sense single-stranded RNA virus and a member of the family *Rhabdoviridae* and genus Nucleorhabdovirus. Maize mosaic virus (MMV) is found in many tropical and subtropical regions in the world, including North America, India, and islands of the Western Indian Ocean (1–4). Virus infection of the host plants, i.e., corn, sorghum, and pearl millet, causes stunting, chlorosis, and grain loss. MMV has an approximately 12-kb genome, with the following six genes arranged 3′ to 5′: nucleoprotein (N), phosphoprotein (P), putative movement protein (3), matrix protein (M), glycoprotein (G), and polymerase protein (L) (5). There is previous sequence information available for MMV; however, the genome was incomplete (5). As MMV is a model for insect transmission of rhabdoviruses (vectored by the corn planthopper [Peregrinus maidis] [6]), there is great interest in completing the genome. The goal of this study is to completely sequence MMV as the first step in creating a full-length infectious clone for insect transmission studies.

The full-length sequence of the MMV genome was generated using two strategies, next-generation RNA sequencing (RNA-Seq) and rapid amplification of cDNA ends (RACE) of the viral genome. This isolate of MMV was originally collected in Hawaii in 1971 and passaged routinely by vector transmission. Briefly, to determine the full-length sequences of MMV, total RNA was isolated from individual insects using the TRIzol reagent, as previously described (7), and each insect was tested for the presence/absence of virus via reverse transcription-PCR (RT-PCR) with previously described primers (8). Three experimental replicates composed of pooled virus-infected or noninfected insects were used for RNA-Seq for a total of six samples. The TruSeq RNA sample preparation kit (Illumina, San Diego, CA) was used to convert the total RNA (2 μg) to cDNA libraries for subsequent cluster generation and sequencing using the manufacturer’s protocols. The six RNA-Seq libraries were single-end sequenced using the HiSeq 2000 platform (Illumina) and TruSeq sequencing by synthesis (SBS) chemistry workflow. A total number of 102,083,710 bases were sequenced, which were assembled into 89,689 components (contigs) with a mean length of 1,138.2 bp (8). Burrows-Wheeler (BWA-MEM) was used to map the raw reads (SRA number PRJNA540525) to those of the MMV genome (GenBank accession number AY618418) (8, 9). The identification of single nucleotide polymorphisms (SNPs) and corrections to the L protein sequence were done using the Integrative Genomics Viewer version 2.3.57. Three missing bases in the L protein gene were identified, as well as 23 SNPs spanning the entire genome. Interestingly, no SNPs were identified in the M sequence. To sequence the missing MMV leader and trailer sequences, we used 5′ and 3′ RACE. Total RNA was extracted from infected corn plants using the Qiagen RNeasy kit, following the kit directions, and this RNA was the template for 5′ and 3′ RACE protocols. To identify the full leader sequences, 3′ RACE of the viral genome was done by adding poly(A) sequences to the leader utilizing Escherichia coli poly(A) polymerase (New England BioLabs, Ipswich, MA) and SuperScript III (Invitrogen, Carlsbad, CA) for first-strand cDNA synthesis and amplification with a poly(T) primer and an N-specific primer (5′-GCAGTCGCCAAATTAGTCCAGTC-3′). This result was confirmed using ligation-anchored 3′ RACE, as described previously (10), using the primers HNEF 5′P-AAGCTTGCGGCCGCGATATC-3′ddC and HNER 5′-GATATCGCGGCCGCAAGCTT-3′. To complete the trailer sequences, we used the Invitrogen (Carlsbad, CA) 5′ RACE system. Once completed and corrected, the total genome of MMV was 12,170 bases long (GenBank accession number MK828539), and the entire genome had ≥10-fold coverage. The leader of MMV is 155 bases long, and the first nine bases are identical to those of taro vein chlorosis virus (TaVCV), 3′-AGAGACCCA-5′ (11). The trailer is 97 bases long, and the terminal 3′ and 5′ ends are complementary, forming a hypothesized “panhandle”-type structure similar to that of other described rhabdoviruses (12). The closest blast hit is the existing MMV genome present in GenBank (accession number AY618418), at 99% similarity. Although there is similarity at the protein level to TaVCV (GenBank accession number AY674964), as previously described (13), the nucleotide similarity of the entire genome is 49%. Recently, the first infectious clone for a nucleorhabdovirus was created (14), and the complete genome sequence of MMV provides the necessary sequence data for developing a similar clone for MMV.

### Data availability.

The data for this paper are available at the NCBI as the completed genome under GenBank accession number MK828539, and the MMV-infected P. maidis RNA-Seq library sequences are under SRA project number PRJNA540525.
